# The First Order Transfer Function in the Analysis of Agrochemical Data in Honey Bees (*Apis Mellifera* L.): Proboscis Extension Reflex (PER) Studies

**DOI:** 10.3390/insects5010167

**Published:** 2014-01-07

**Authors:** Lisa A. De Stefano, Igor I. Stepanov, Charles I. Abramson

**Affiliations:** 1 Laboratory of Comparative Psychology and Behavioral Biology, Oklahoma State University, Stillwater, OK 74078, USA; E-Mail: lisa.destefano@gmail.com; 2 Department of Neuropharmacology, Institute for Experimental Medicine, 12 Acad. Pavlov Street, St. Petersburg 197376, Russia; E-Mail: igorstep@is12044.spb.edu

**Keywords:** mathematical model, agrochemicals, proboscis conditioning in honey bees

## Abstract

This paper describes a mathematical model of the learning process suitable for studies of conditioning using the proboscis extension reflex (PER) in honey bees when bees are exposed to agrochemicals. Although procedural variations exist in the way laboratories use the PER paradigm, proboscis conditioning is widely used to investigate the influence of pesticides and repellents on honey bee learning. Despite the availability of several mathematical models of the learning process, no attempts have been made to apply a mathematical model to the learning curve in honey bees exposed to agrochemicals. Our model is based on the standard transfer function in the form Y = B3e^−B2 (X−1)^ + B4 (1−e^−B2 (X−1)^) where X is the trial number, Y is the proportion of correct responses, B2 is the learning rate, B3 is readiness to learn, and B4 is ability to learn. We reanalyze previously published data on the effect of several classes of agrochemicals including: (1) those that are considered harmless to bees (e.g., pymetrozine, essential oils, dicofol); (2) sublethal exposure to pesticides known to harm honey bees (e.g., coumaphos, cyfluthrin, fluvalinate, permethrin); and (3) putative repellents of honey bees (e.g., butyric acid, citronella). The model revealed additional effects not detected with standard statistical tests of significance.

## 1. Introduction

The application of mathematical models in the analysis of learning data has a rich tradition in experimental psychology. Mathematical models of the learning process have been developed for various phenomena including maze learning, avoidance behavior, classical conditioning and operant conditioning. For readers interested in the history of mathematical models in experimental psychology, consult Luce, Bush, and Galanter [1] and Stepanov and Abramson [2]. Much information is also available from the Society for the Quantitative Analyses of Behavior [3].

As Mazur [4] suggests, the use of mathematical models in the analysis of behavior has much to recommend it. Models of the learning process conveniently summarize large amounts of data, guide research and theory, and assist researchers in defining terms. Mathematical models help researchers integrate factual information into theories of the learning process. We would also add that the use of models stimulates collaborative research among experimental psychologists and those scientists concerned with agricultural issues. The study of learning has been an integral part of experimental psychology since its founding in 1879, but few psychologists appear to be involved in agricultural research [5].

We believe that the lack of collaboration among psychologists and those interested in agricultural research is unfortunate. This is most readily seen in the lack of mathematical models applied to the behavioral effects of agrochemicals. The use of such models can characterize the results of various agrochemical treatments across laboratories, species, and conditions. It can also identify weaknesses in research designs that cloud data interpretation, and provide important information above and beyond that provided by standard significance testing.

Although procedural variations exist in the way laboratories use the proboscis extension reflex (PER) paradigm and there is some discussion about replication and usefulness of the technique [6,7], the PER is widely used to investigate the influence of pesticides and repellents on honey bee learning [8]. In the PER paradigm, harnessed bees receive odor (the conditioned stimulus or CS) and sucrose (the unconditioned stimulus or US) pairings. After several CS-US pairings, the proboscis extends to the CS prior to the presentation of the US. Honey bees have been exposed to agrochemicals by embedding them in the CS and US [9,10], or during a pretreatment phase [11].

Surprisingly, only one previous attempt to mathematically characterize the learning process following exposure to agrochemicals has been reported. Abramson and Stepanov [12] studied the effect of insect growth regulators tebufenozide (Confirm^®^2F) and diflubenzuron (Dimilin^®^) on the classical conditioning of proboscis extension. The model revealed that the main effect of Confirm^®^2F was to decrease the learning rate. In contrast, the main effect of Dimilin^®^ was to decrease the ability to learn. These results suggest that Dimilin^®^ is more dangerous to honey bees than Confirm^®^2F even though both insect growth regulators are considered “harmless” to honey bees.

The purpose of the present study is to extend our earlier modeling efforts by reanalyzing previously published data on the effect of several classes of agrochemicals including: (1) those that are considered harmless to bees (e.g., pymetrozine, essential oils, dicofol); (2) sublethal exposure to pesticides known to harm honey bees (e.g., coumaphos, cyfluthrin, fluvalinate, permethrin); and (3) putative repellents of honey bees (e.g., butyric acid, citronella).

## 2. Experimental Section

### 2.1. Selection of Articles for Modeling

We used the following criteria for selecting the articles used in the analysis.

First, there must be a minimum of six training trials. With fewer than six training trials, we believe that it is impossible to accurately use any mathematical models [2]. Six training trials may not be enough to reveal stable performance in animals pre-exposed to an agrochemical or if the agrochemical is imbedded in the unconditioned stimulus or reward. Some agrochemicals might decrease the learning rate, so that the learning curve is linear rather than exponential. In our model, this is evident when a low B2 value is matched to an extremely high B4 value. Such a high B4 value, though being mathematically correct, is far from being biologically plausible. Moreover, an agrochemical may exert its effect after six trials. A search of the literature revealed that nearly all of the agrochemical studies that used more than six training trials were those from the senior author’s laboratory. In an effort to increase the number of studies that we can apply the model to, we accepted studies that used five training trials and copied the data from the fifth training trial to create a “sixth” training trial. This additional trial is shown in Table 1 in italics. In our laboratory, we use 12 training trials.

Second, we focused on studies that present the conditioning data in the form of trial by trial learning curves. This was done because our model is based on a learning curve. Third, we tried to select studies that used control groups either in the form of unpaired presentations of the CS and US, or, in the case of within group experimental designs, discrimination procedures in which one CS was paired with a feeding and a second CS was not. Without such control groups, it is difficult to make the claim that exposure to agrochemicals does indeed influence learning [13]. It is equally plausible the effect is on non-associative processes such as habituation and/or sensitization. It was not always possible to find experiments from different laboratories that employ unpaired or discrimination control groups. For the sake of diversity, we included such experiments if they presented data in the form of learning curves. Fourth, we only focused on acquisition and not extinction. Our model was developed to focus on the acquisition of learning. Fifth, we selected articles that offered a range of agrochemicals, including repellents, sublethal effects of agrochemicals, and agrochemicals considered safe for honey bees.

Information about the amount of agrochemical given to honey bees, as well as general information about the experimental procedure, is available in the brief introduction to each experiment (Section 3). Specific details of individual procedures are available in the original citations. It should be noted that PER methodology is not standard between laboratories. In the studies from the senior author’s laboratory, the CS duration is 3 s, and the US duration is 2 s. We also use a non-overlap procedure in which the CS terminates before the US is presented.

**Table 1:** Raw bee learning data. The raw data are presented to aid researchers who wish to apply other models of the learning process, as well as to provide context for the B2, B3 and B4 values. The source listed is the original source of the data, and can be found in the references.

No	Source	Experiment/chemicals	Trial 1	Trial 2	Trial 3	Trial 4	Trial 5	Trial 6	Trial 7	Trial 8	Trial 9	Trial 10	Trial 11	Trial 12
1.	[14]	a pretreatment: a field dose of pymetrozine	0	5	25	35	50	55	50	60	60	60	55	55
2.	[14]	a pretreatment: 100× the field dose of pymetrozine	5	5	0	10	15	15	15	15	15	15	10	10
3.	[14]	a pretreatment: sucrose only	5	50	65	60	70	75	75	70	75	70	75	75
4.	[14]	a pretreatment: a field dose of pymetrozine	10	20	25	40	70	55	70	50	50	60	65	70
5.	[14]	a pretreatment: 100× the field dose of pymetrozine	0	25	20	25	30	15	15	15	5	5	0	0
6.	[14]	control, sucrose only	0	45	70	65	80	75	85	90	85	85	85	85
7.	[9]	a US: 6.25% Bioganic	10	5	0	0	5	0	0	0	0	0	5	0
8.	[9]	a US: 1.56% Bioganic	10	25	50	50	35	30	35	20	20	20	20	15
9.	[9]	a US: sucrose only	0	25	45	65	60	65	65	60	70	55	60	65
10.	[9]	a US: 6.25% Bioganic	10	5	5	10	0	0	0	0	0	0	0	0
11.	[9]	a US: 1.56% Bioganic	5	10	25	40	20	0	10	5	25	5	5	10
12.	[9]	a US: sucrose only	10	60	70	75	70	90	75	70	75	70	75	75
13.	[9]	a CS: citronella odor	0	25	60	70	70	70	75	70	75	75	80	80
14.	[9]	a CS: 6.25% Bioganic odor	30	60	75	80	80	90	85	80	85	80	85	85
15.	[15]	a CS+: pignut	10	60	85	95	95	95	95	90	95	90	85	85
16.	[15]	a CS+: sweet fennel	10	75	85	85	75	75	80	75	85	85	85	80
17.	[16]	a pretreatment: 10 μL dicofol	5	30	45	60	55	50	60	55	55	55	55	55
18.	[16]	a pretreatment: sucrose	20	70	75	80	75	80	90	75	90	75	80	75
19.	[16]	a pretreatment: 10 μL dicofol	10	55	65	75	80	70	–	–	–	–	–	–
20.	[16]	a pretreatment: sucrose	15	30	60	75	80	75	–	–	–	–	–	–
21.	[10]	a CS: butyric acid	5	30	70	75	75	85	90	85	90	85	85	85
22.	[10]	a CS: DEET	15	65	75	80	75	75	70	75	75	70	75	70
23.	[10]	a CS: cinnamon	10	50	70	85	85	85	95	95	100	90	100	95
24.	[17]	a CS: citronella	5	30	55	50	55	60	55	60	65	60	55	55
25.	[17]	a CS: cinnamon	5	20	53	53	53	53	58	58	63	63	63	70
26.	[18]	a pretreatment: fluvalinate	38.5	52.6	58.8	62.2	65.7	67.7	72.7	–	–	–	–	–
27.	[18]	a pretreatment: acetone	62.9	76.4	83.9	90.3	91.7	90.5	89.6	–	–	–	–	–
28.	[18]	a pretreatment: flucythrinate	24.2	32.4	36.1	44.0	36.1	42.9	47.2	–	–	–	–	–
29.	[18]	a pretreatment: cyfluthrin	2.9	25.0	33.0	53.1	60.0	59.4	60.0	–	–	–	–	–
30.	[18]	a pretreatment: cypermethrin	34.2	40.0	52.8	58.3	67.7	57.6	62.9	–	–	–	–	–
31.	[18]	a pretreatment: permethrin	41.7	47.5	65.8	59.5	61.1	61.1	67.7	–	–	–	–	–
32.	[18]	a pretreatment: fenvalerate	26.7	41.2	53.1	55.9	66.7	59.4	69.7	–	–	–	–	–
33.	[19]	a pretreatment: water	0	20	40	45	55	55	–	–	–	–	–	–
34.	[19]	a pretreatment: acetone	0	20	50	55	60	60	–	–	–	–	–	–
35.	[19]	a pretreatment: 0.01% coumaphos	0	15	60	65	60	60	–	–	–	–	–	–
36.	[19]	a pretreatment: 0.1% coumaphos	0	30	50	57	70	79	–	–	–	–	–	–
37.	[19]	a pretreatment: 10% coumaphos	0	15	35	55	60	60	–	–	–	–	–	–
38.	[19]	a pretreatment: water	0	20	35	55	60	60	–	–	–	–	–	–
39.	[19]	a pretreatment: acetone	0	20	45	50	60	60	–	–	–	–	–	–
40.	[19]	a pretreatment: 0.005% diazinon	0	20	55	55	60	60	–	–	–	–	–	–
41.	[19]	a pretreatment: 0.01% diazinon	0	20	35	43	55	55	–	–	–	–	–	–
42.	[19]	a pretreatment: 0.025% diazinon	0	45	70	78	78	78	–	–	–	–	–	–
43.	[19]	a pretreatment: hexane only	0	25	45	63	70	70	–	–	–	–	–	–
44.	[19]	a pretreatment: 0.005% diazinon	5	17	25	25	30	30	–	–	–	–	–	–
45.	[19]	a pretreatment: 0.07% coumaphos	0	50	60	67	65	65	–	–	–	–	–	–
46.	[20]	a pretreatment: sucrose	10	5	15	35	55	50	65	65	65	70	60	70
47.	[20]	a pretreatment: endosulfan	0	0	10	0	0	10	15	5	0	0	0	0
48.	[20]	a pretreatment: decis	0	10	10	15	30	40	45	50	45	45	45	40
49.	[20]	a pretreatment: baytroid	0	0	0	0	0	0	0	0	0	0	0	0
50.	[20]	a pretreatment: sevin	0	0	0	0	0	0	0	0	0	0	0	0
51.	[20]	a CS: hexanal	5	20	20	25	35	45	50	70	70	65	75	70
52.	[20]	a CS: endosulfan	5	10	30	35	40	50	60	65	55	65	65	65
53.	[20]	a CS: decis	10	10	20	25	35	50	55	50	60	65	70	60
54.	[20]	a CS: baytroid	0	10	25	25	40	50	50	45	60	55	65	65
55.	[20]	a CS: sevin	5	10	15	30	40	55	65	65	60	70	60	75
56.	[20]	a US: sucrose	5	5	15	25	40	55	55	60	60	60	65	70
57.	[20]	a US: endosulfan	5	15	40	45	60	45	55	45	25	20	15	15
58.	[20]	a US: decis	5	10	20	25	25	45	50	50	55	50	55	50
59.	[20]	a US: baytroid	10	10	5	10	5	5	0	0	0	0	0	0
60.	[20]	a US: sevin	5	15	15	10	15	0	0	0	0	0	0	0
61.	[11]	a pretreatment: 0 μg tebufenozide	12	80	92	91	96	100	92	88	100	92	84	88
62.	[11]	a pretreatment: 16 μg tebufenozide	4	56	84	88	88	92	88	92	92	88	88	88
63.	[11]	a pretreatment: 24 μg tebufenozide	8	32	48	52	48	68	63	52	60	72	76	76
64.	[11]	a pretreatment: 32 μg tebufenozide	4	24	44	44	44	48	68	48	52	68	64	80
65.	[11]	a pretreatment: 69.4 μg tebufenozide	24	48	60	76	76	84	80	80	96	96	88	100
66.	[11]	a pretreatment: 131 μg tebufenozide	8	32	52	48	48	56	56	44	64	64	52	64
67.	[11]	a US: 0 μg tebufenozide	4	52	76	92	88	84	84	84	84	84	84	84
68.	[11]	a US: 16 μg tebufenozide	0	40	64	60	68	80	68	64	64	56	60	64
69.	[11]	a US: 24 μg tebufenozide	4	28	48	56	52	64	64	60	64	56	60	60
70.	[11]	a US: 32 μg tebufenozide	8	32	36	52	72	64	88	64	68	64	64	68
71.	[11]	a US: 69.4 μg tebufenozide	4	20	44	48	52	56	68	64	64	56	60	56
72.	[11]	a US: 131 μg tebufenozide	8	36	60	60	60	76	60	68	72	76	72	72
73.	[11]	a pretreatment: 0 μg diflubenzuron	0	60	76	80	84	92	80	84	76	80	80	84
74.	[11]	a pretreatment: 3.4 μg diflubenzuron	8	32	52	68	60	56	64	72	60	64	64	68
75.	[11]	a pretreatment: 8.5 μg diflubenzuron	12	44	36	68	52	56	64	72	72	68	72	80
76.	[11]	a pretreatment: 16 μg diflubenzuron	16	52	48	76	76	84	84	76	84	76	76	68
77.	[11]	a pretreatment: 32 μg diflubenzuron	12	44	48	60	60	72	64	64	52	64	52	48
78.	[11]	a pretreatment: 69.4 μg diflubenzuron	16	36	28	44	44	48	48	52	52	48	56	56
79.	[11]	a US: 0 μg diflubenzuron	12	48	52	60	56	56	68	60	52	52	60	64
80.	[11]	a US: 3.4 μg diflubenzuron	20	28	28	36	36	28	32	24	24	32	28	24
81.	[11]	a US: 8.5 μg diflubenzuron	4	24	48	36	32	36	32	36	32	32	28	16
82.	[11]	a US: 16 μg diflubenzuron	28	16	28	28	44	24	32	40	44	44	40	32
83.	[11]	a US: 32 μg diflubenzuron	8	24	40	52	56	40	24	28	28	24	20	28
84.	[11]	a US: 69.4 μg diflubenzuron	28	40	56	32	32	24	16	32	28	28	24	16
85.	[11]	a pretreatment: 0 μg tebufenozide	10	58	81	88	77	77	77	77	77	81	77	73
86.	[11]	a pretreatment: 16 μg tebufenozide	10	30	58	77	73	73	69	77	73	65	65	65
87.	[11]	a pretreatment: 24 μg tebufenozide	7	37	42	54	65	73	69	69	73	81	77	85
88.	[11]	a pretreatment: 32 μg tebufenozide	7	26	26	26	58	58	61	65	50	60	50	58
89.	[11]	a pretreatment: 69.4 μg tebufenozide	10	46	58	65	65	73	65	69	73	69	58	73
90.	[11]	a pretreatment: 131 μg tebufenozide	3	30	42	54	54	38	50	54	54	58	69	60
91.	[11]	a US: 0 μg tebufenozide	3	65	69	84	84	77	81	88	81	81	84	81
92.	[11]	a US: 16 μg tebufenozide	10	42	64	54	57	50	77	73	58	54	69	61
93.	[11]	a US: 24 μg tebufenozide	7	37	57	73	58	77	50	61	49	41	53	45
94.	[11]	a US: 32 μg tebufenozide	10	26	34	38	45	50	60	58	58	58	50	58
95.	[11]	a US: 69.4 μg tebufenozide	7	27	53	54	64	53	61	61	60	58	53	53
96.	[11]	a US: 131 μg tebufenozide	14	34	41	57	54	53	64	58	58	58	46	42
97.	[11]	a pretreatment: 0 μg diflubenzuron	15	15	27	42	46	42	38	42	38	46	58	54
98.	[11]	a pretreatment: 3.4 μg diflubenzuron	15	38	61	54	54	69	65	62	69	58	50	62
99.	[11]	a pretreatment: 8.5 μg diflubenzuron	11	31	27	42	58	61	65	62	69	58	65	69
100.	[11]	a pretreatment: 16 μg diflubenzuron	4	34	50	54	62	58	58	54	46	46	50	62
101.	[11]	a pretreatment: 32 μg diflubenzuron	7	31	54	54	42	50	62	62	50	65	77	58
102.	[11]	a pretreatment: 69.4 μg diflubenzuron	15	34	46	50	62	58	58	65	57	50	54	46
103.	[11]	a US: 0 μg diflubenzuron	4	19	35	38	39	46	35	58	50	50	50	54
104.	[11]	a US: 3.4 μg diflubenzuron	0	8	19	23	23	27	27	23	27	23	31	35
105.	[11]	a US: 8.5 μg diflubenzuron	4	27	34	23	23	27	27	23	27	27	31	31
106.	[11]	a US: 16 μg diflubenzuron	23	23	31	19	35	31	42	38	39	42	38	39
107.	[11]	a US: 32 μg diflubenzuron	19	12	23	42	31	31	31	31	23	35	27	27
108.	[11]	a US: 69.4 μg diflubenzuron	4	19	42	42	31	27	27	27	11	15	23	11

### 2.2. Obtaining Data for Analysis

Because it is often difficult to obtain raw data, we decided to gather data by extracting it from published graphs. We tried several software packages and came to the conclusion that the free web-based program “WebPlotDigitizer” Version 2.5 fit our needs [21]. The program uses HTML5, so it can run within a browser. For each of the experiments selected for analysis, we:

1. Copied the graph(s) of interest from a published manuscript.
2. Entered the graph into the “WebPlotDigitizer” software.
3. Copied the data into an Excel spreadsheet.
4. Entered the data into the “Learning Curve Modeling Tool” program to obtain the model’s coefficients.
5. Compiled the data, organized by author/agrochemical/year.
6. Tested the significance of the model’s coefficients using SPSS and Mathematica.

### 2.3. Estimation of the Model’s Parameters and Its Verification

SPSS and Mathematica were used to estimate the model’s parameters. R squared (R^2^) was used for verification. The closer the learning data are to the model values, the higher the R^2^, its maximal value being equal to 1. A step-by-step guide to making the calculations using SPSS or Mathematica is provided in a recent paper [22]. To make the calculations as easy as possible, we have developed software that is available upon request (The Learning Curve Modeling Tool or LCMT).

### 2.4. Description of the Model

The model has been described in detail in several previous publications [2,22,23]. It is based on the first order system transfer function in the form Y = B3e^−B2 (X−1)^ + B4 (1−e^−B2 (X−1)^) where X is the trial number and Y is the proportion of correct responses. The model contains three parameters: B2, the learning rate; B4, the asymptotic value of correct responses at X = Infinity; and B3, the value of correct responses at the beginning of training (*i.e*., B3 = Y at X = 1). Because B2 = 1/τ, it means that 1/B2 is the number of trials required for achievement of 63% from the difference between B3 and B4.

B3 is considered to be an estimate of the functional state of an animal at the onset of training that accounts for, in particular, the value of prior learning based on previous experience and readiness to learn. Because the independent variable is the value of correct responses (*i.e*., a proboscis extension to the CS), the greater the value of B3, the higher is the “readiness to learn”.

The learning mechanisms are reflected in the learning rate (B2) and asymptotic level (B4). In the case of PER conditioning, B4 is the maximum possible number of correct responses after a very large number of trials. The greater the value of B4, the higher is the ability to learn. Our rationale for using coefficient B4 is that the number of correct responses does not always increase to 1 (or 100%). That is why, in our view, it is impossible to pre-fix B4 to 1.0. Below, B4 is designated the “ability to learn.”

Additionally, the value of coefficient B2 is restricted. By definition of the mathematical model, coefficient B2 must exceed zero. The upper limit that has been established for B2 is equal to 5.0. It is known that the first order system reaches 99.3% of its asymptotic value during five time constants [24,25]. Because B2 = 1/τ, it means that if B2 = 5, then an animal achieves its asymptotic maximum of conditioned responses in one session. In other words, we suppose that the exponential learning curve exists, if 0.001 < B2 < 5.0.

## 3. Results and Discussion

Table 1 provides the raw data. We provide the raw data as a convenience to other researchers who wish to test other models of the learning process. The model’s coefficients are given in Table 2. Significance levels for comparison of the model’s coefficients are presented in Table 3.

### 3.1. Agrochemicals Considered Harmless to Bees

*Pymetrozine* (Plenum WG-50^®^). This agrochemical is a systemic pesticide of the pyridine-azomethin family and is considered harmless to bees. In this experiment, a non-overlap procedure was used with a CS duration of 3 s, a US duration of 2 s, and an intertrial interval of 10 min. Forager honey bees *Apis mellifera*: Hybrid var. Buckfast were used. The bees were collected in glass vials at the laboratory feeder approximately 24 h prior to use. The vials were placed in an ice water bath to reduce activity and then placed in a harness, fed 1.8 M sucrose, and set aside. The following day, each bee received a pre-test in which the antennae was stimulated with sucrose. If a vigorous proboscis response was observed, the bee was used 10 min or more later. The 10 min delay was used to reduce the excitation induced by sucrose stimulation. The agrochemical was administered orally and mixed with sucrose to make it palatable. Two doses were used. In one, the recommend field dose was used (5 μL of 0.3 g L^−1^, 0.16 g L^−1^ of pymetrozine) and in the other, 100× the recommended field dose was used (5 μL of 30 g L^−1^, 0.14 g L^−1^ of pymetrozine) [14].

Experiment 1 investigated the effects of pymetrozine on simple Pavlovian conditioning where honey bees were trained to associate a CS with a US. The control group without the chemical learned well, with more than 70% of bees responding (Table 1, item 3). The model fit the learning data well; R squared is about 0.97, and ability to learn (B4) is about 73% (Table 2, item 3). A pretreatment with a field dose of pymetrozine decreased the learning rate (B2) three times in comparison with the control group (Table 3, item 2); other coefficients did not differ. A pretreatment with 100× the field dose of pymetrozine practically prevented learning, having lowered the number of conditioned responses (CR) to 15% with a further decrease on trials 11 and 12, so that the fit was much worse in comparison with the control group as well as the group pretreated with a field dose (Table 2, item 2). Ability to learn was significantly lower in comparison with the control group (Table 3, item 3) as well as with the field dose group (Table 3, item 1).

**Table 2:** Learning curve modeling. The data from Table 1 are analyzed below and coefficients are listed for B2, B3 and B4. The source listed is the original source of the data, and can be found in the references.

No	Source	Experiment/chemicals	Model’s coefficients (with standard error)			R squared
			B2	B3	B4	
1.	[14]	a pretreatment: a field dose of pymetrozine	0.30 ± 0.06	0.0 ± 11.0	63.0 ± 4.4	0.90
2.	[14]	a pretreatment: 100× the field dose of pymetrozine	0.35 ± 0.31	2.3 ± 3.6	14.0 ± 2.8	0.44
3.	[14]	a pretreatment: sucrose only	0.97 ± 0.14	6.0 ± 3.9	72.7 ± 1.4	0.97
4.	[14]	a pretreatment: a field dose of pymetrozine	0.34 ± 0.15	5.9 ± 9.4	66.1 ± 7.6	0.80
5.	[14]	a pretreatment: 100× the field dose of pymetrozine	The data are not fitted with the model.			
6.	[14]	control, sucrose only	0.70 ± 0.09	1.0 ± 4.8	85.0 ± 2.0	0.98
7.	[9]	a US: 6.25% Bioganic	1.22 ± 0.81	10.2 ± 2.3	1.0 ± 0.8	0.70
8.	[9]	a US: 1.56% Bioganic	2.73 ± 10.75	9.9 ± 12.9	29.2 ± 4.1	0.19
9.	[9]	a US: sucrose only	0.67 ± 0.12	0.0 ± 12	64.0 ± 2.2	0.97
10.	[9]	a US: 6.25% Bioganic	0.25 ± 0.19	9.1 ± 2.2	−1.2 ± 2.7	0.41
11.	[9]	a US: 1.56% Bioganic	The data are not fitted with the model.			
12.	[9]	a US: sucrose only	1.45 ± 0.38	10.1 ± 5.8	75.0 ± 2.0	0.94
13.	[9]	a CS: citronella odor	0.60 ± 0.08	0.0 ± 10.4	76.9 ± 2.1	0.96
14.	[9]	a CS: 6.25% Bioganic odor	0.86 ± 0.12	29.8 ± 3.0	83.9 ± 1.2	0.98
15.	[15]	a CS+: pignut	1.10 ± 0.16	9.1 ± 4.8	92.1 ± 1.7	1.00
16.	[15]	a CS+: sweet fennel	2.61 ± 0.94	10.0 ± 4.7	80.9 ± 1.5	0.97
17.	[16]	a pretreatment: 10 μL dicofol	0.83 ± 0.15	4.0 ± 3.8	56.0 ± 1.5	0.95
18.	[16]	a pretreatment: sucrose	1.64 ± 0.50	20.1 ± 5.7	80.0 ± 1.9	0.99
19.	[16]	a pretreatment: 10 μL dicofol	1.12 ± 0.26	10.1 ± 4.9	75.3 ± 3.3	0.98
20.	[16]	a pretreatment: sucrose	0.44 ± 0.22	11.6 ± 7.8	90.2 ± 16.6	0.95
21.	[10]	a CS: butyric acid	0.59 ± 0.09	2.0 ± 5.3	87.6 ± 2.4	0.96
22.	[10]	a CS: DEET	1.99 ± 0.41	14.6 ± 3.2	74.1 ± 1.1	0.99
23.	[10]	a CS: cinnamon	0.60 ± 0.06	10.1 ± 3.8	95.7 ± 1.7	0.99
24.	[17]	a CS: citronella	0.79 ± 0.15	4.7 ± 4.4	58.6 ± 1.7	0.96
25.	[17]	a CS: cinnamon	0.52 ± 0.12	4.1 ± 5.5	63.1 ± 2.8	0.91
26.	[18]	a pretreatment: fluvalinate	0.41 ± 0.09	39.4 ± 1.8	73.5 ± 3.0	0.98
27.	[18]	a pretreatment: acetone	0.71 ± 0.13	62.5 ± 1.7	91.8 ± 1.4	0.98
28.	[18]	a pretreatment: flucythrinate	0.39 ± 0.30	24.6 ± 3.7	46.7 ± 6.8	0.84
29.	[18]	a pretreatment: cyfluthrin	0.41 ± 0.12	2.2 ± 4.4	68.6 ± 7.4	0.84
30.	[18]	a pretreatment: cypermethrin	0.45 ± 0.26	32.4 ± 4.8	66.2 ± 7.1	0.88
31.	[18]	a pretreatment: permethrin	0.64 ± 0.43	40.8 ± 5.1	65.0 ± 4.5	0.81
32.	[18]	a pretreatment: fenvalerate	0.42 ± 0.16	26.6 ± 3.9	70.4 ± 6.2	0.95
33.	[19]	a pretreatment: water	0.37 ± 0.13	−0.1 ± 3.1	70.0 ± 13.1	0.99
34.	[19]	a pretreatment: acetone	0.46 ± 0.25	0.0 ± 6.4	74.1 ± 18.8	0.97
35.	[19]	a pretreatment: 0.01% coumaphos	0.51 ± 0.50	0.0 ± 15.2	77.5 ± 34.2	0.89
36.	[19]	a pretreatment: 0.1% coumaphos	0.45 ± 0.1	0.3 ± 2.8	82.0 ± 8.9	0.99
37.	[19]	a pretreatment: 10% coumaphos	0.51 ± 0.43	0.0 ± 9.5	63.9 ± 23.5	0.93
38.	[19]	a pretreatment: water	0.53 ± 0.35	0.3 ± 7.7	63.4 ± 17.6	0.95
39.	[19]	a pretreatment: acetone	0.37 ± 0.17	−0.6 ± 4.3	78.1 ± 19.0	0.98
40.	[19]	a pretreatment: 0.005% diazinon	0.52 ± 0.36	0.0 ± 8.9	71.4 ± 21.6	0.94
41.	[19]	a pretreatment: 0.01% diazinon	0.52 ± 0.23	0.3 ± 4.5	57.3 ± 10.8	0.98
42.	[19]	a pretreatment: 0.025% diazinon	0.84 ± 0.12	−0.1 ± 3	83.1 ± 3.6	1.00
43.	[19]	a pretreatment: hexane only	0.57 ± 0.27	0.2 ± 6.9	73.4 ± 14.3	0.97
44.	[19]	a pretreatment: 0.005% diazinon	0.61 ± 0.21	5.0 ± 1.9	31.4 ± 3.5	0.98
45.	[19]	a pretreatment: 0.07% coumaphos	1.42 ± 0.15	−0.01 ± 1.9	65.8 ± 1.4	1.00
46.	[20]	a pretreatment: sucrose	0.21 ± 0.08	−0.7 ± 7.2	79.0 ± 12.1	0.90
47.	[20]	a pretreatment: endosulfan	1.26 ± 4.68	−0.6 ± 5.8	3.7 ± 2.0	0.05
48.	[20]	a pretreatment: decis	0.24 ± 0.09	0.0 ± 12.0	52.0 ± 7.8	0.89
49.	[20]	a pretreatment: baytroid	There is no data because learning did not occur.			
50.	[20]	a pretreatment: sevin	There is no data because learning did not occur.			
51.	[20]	a CS: hexanal	0.19 ± 0.09	5.3 ± 7.0	78.2 ± 14	0.91
52.	[20]	a CS: endosulfan	0.22 ± 0.05	1.7 ± 4.1	73.2 ± 6.3	0.96
53.	[20]	a CS: decis	0.18 ± 0.09	10 ± 6.1	72.5 ± 14.6	0.91
54.	[20]	a CS: baytroid	0.18 ± 0.05	−0.6 ± 4.0	74.8 ± 9.3	0.96
55.	[20]	a CS: sevin	0.17 ± 0.06	0.0 ± 12.5	85.8 ± 14.6	0.93
56.	[20]	a US: sucrose	0.16 ± 0.05	0.0 ± 4.8	85.2 ± 14.0	0.95
57.	[20]	a US: endosulfan	1.18 ± 1.61	2.9 ± 10.3	35.9 ± 6.0	0.28
58.	[20]	a US: decis	0.18 ± 0.07	1.4 ± 4.6	64.5 ± 10.1	0.93
59.	[20]	a US: baytroid	0.26 ± 1.03	10.0 ± 2.5	3.9 ± 13.6	0.45
60.	[20]	a US: sevin	1.91 ± 3.11	5.0 ± 3.4	14.1 ± 2.3	0.70
61.	[11]	a pretreatment: 0 μg tebufenozide	1.89 ± 0.45	12.1 ± 5.2	92.4 ± 1.7	0.96
62.	[11]	a pretreatment: 16 μg tebufenozide	1.05 ± 0.08	3.5 ± 2.7	90.1 ± 1.0	0.99
63.	[11]	a pretreatment: 24 μg tebufenozide	0.36 ± 0.12	11.0 ± 6.9	70.6 ± 5.1	0.88
64.	[11]	a pretreatment: 32 μg tebufenozide	0.25 ± 0.12	9.0 ± 8.0	71.5 ± 10.1	0.84
65.	[11]	a pretreatment: 69.4 μg tebufenozide	0.35 ± 0.07	26.0 ± 5.0	94.9 ± 3.8	0.95
66.	[11]	a pretreatment: 131 μg tebufenozide	0.71 ± 0.23	8.6 ± 6.8	57.1 ± 2.8	0.84
67.	[11]	a US: 0 μg tebufenozide	1.04 ± 0.13	3.0 ± 4.1	85.5 ± 1.5	0.98
68.	[11]	a US: 16 μg tebufenozide	1.12 ± 0.3	−0.7 ± 6.8	65.5 ± 2.4	0.91
69.	[11]	a US: 24 μg tebufenozide	0.66 ± 0.1	3.0 ± 3.6	61.2 ± 1.6	0.96
70.	[11]	a US: 32 μg tebufenozide	0.49 ± 0.16	6.1 ± 8.7	70.4 ± 4.6	0.85
71.	[11]	a US: 69.4 μg tebufenozide	0.510.1	1.8 ± 4.8	61.8 ± 2.4	0.94
72.	[11]	a US: 131 μg tebufenozide	0.64 ± 0.13	7.9 ± 5.3	71.0 ± 2.3	0.94
73.	[11]	a pretreatment: 0 μg diflubenzuron	1.3 ± 0.19	0.0 ± 4.3	82.4 ± 1.5	0.97
74.	[11]	a pretreatment: 3.4 μg diflubenzuron	0.72 ± 0.15	6.6 ± 5.3	65.2 ± 2.2	0.93
75.	[11]	a pretreatment: 8.5 μg diflubenzuron	0.34 ± 0.13	16.8 ± 7.5	74.4 ± 6.1	0.85
76.	[11]	a pretreatment: 16 μg diflubenzuron	0.67 ± 0.19	16.2 ± 7.4	78.6 ± 3.2	0.88
77.	[11]	a pretreatment: 32 μg diflubenzuron	1.03 ± 0.4	12.1 ± 7.3	59.5 ± 2.6	0.81
78.	[11]	a pretreatment: 69.4 μg diflubenzuron	0.31 ± 0.1	18.4 ± 3.8	55.4 ± 3.5	0.90
79.	[11]	a US: 0 μg diflubenzuron	1.35 ± 0.43	12.3 ± 5.2	58.6 ± 1.8	0.89
80.	[11]	a US: 3.4 μg diflubenzuron	2.06 ± 4.15	20.2 ± 4.6	29.3 ± 1.5	0.28
81.	[11]	a US: 8.5 μg diflubenzuron	1.99 ± 2.17	3.7 ± 8.2	32.3 ± 2.7	0.55
82.	[11]	a US: 16 μg diflubenzuron	0.16 ± 0.28	21.9 ± 6.3	43.7 ± 17.1	0.43
83.	[11]	a US: 32 μg diflubenzuron	1.69 ± 2.7	7.9 ± 12.6	33.7 ± 4.2	0.30
84.	[11]	a US: 69.4 μg diflubenzuron	The data are not fitted with the model.			
85.	[11]	a pretreatment: 0 μg tebufenozide	1.46 ± 0.31	9.4 ± 4.9	78.5 ± 1.7	0.95
86.	[11]	a pretreatment: 16 μg tebufenozide	0.77 ± 0.2	6.8 ± 7.2	71.2 ± 2.9	0.89
87.	[11]	a pretreatment: 24 μg tebufenozide	0.34 ± 0.06	9.7 ± 4.2	81.0 ± 3.3	0.97
88.	[11]	a pretreatment: 32 μg tebufenozide	0.36 ± 0.15	5.7 ± 8.3	60.2 ± 6.2	0.81
89.	[11]	a pretreatment: 69.4 μg tebufenozide	0.91 ± 0.18	10.1 ± 4.6	68.3 ± 1.7	0.94
90.	[11]	a pretreatment: 131 μg tebufenozide	0.61 ± 0.2	4.5 ± 7.2	56.6 ± 3.2	0.85
91.	[11]	a US: 0 μg tebufenozide	1.34 ± 0.2	3.6 ± 4.2	81.8 ± 1.4	0.97
92.	[11]	a US: 16 μg tebufenozide	1.05 ± 0.47	10.1 ± 9.2	62.3 ± 3.3	0.77
93.	[11]	a US: 24 μg tebufenozide	1.35 ± 0.89	6.0 ± 11.7	56.4 ± 4.0	0.65
94.	[11]	a US: 32 μg tebufenozide	0.35 ± 0.08	10.3 ± 3.8	58.6 ± 2.8	0.94
95.	[11]	a US: 69.4 μg tebufenozide	0.8 ± 0.18	5.0 ± 5.1	58.5 ± 2.0	0.92
96.	[11]	a US: 131 μg tebufenozide	0.79 ± 0.32	13.3 ± 7.0	54.5 ± 2.7	0.79
97.	[11]	a pretreatment: 0 μg diflubenzuron	0.26 ± 0.13	12.8 ± 5.7	52.6 ± 7.0	0.80
98.	[11]	a pretreatment: 3.4 μg diflubenzuron	0.9 ± 0.32	14.5 ± 6.8	61.2 ± 2.6	0.83
99.	[11]	a pretreatment: 8.5 μg diflubenzuron	0.3 ± 0.08	10.4 ± 5.3	70 ± 5.0	0.92
100.	[11]	a pretreatment: 16 μg diflubenzuron	1.09 ± 0.32	3.2 ± 5.8	54.4 ± 2.1	0.89
101.	[11]	a pretreatment: 32 μg diflubenzuron	0.55 ± 0.22	9.0 ± 8.8	61.0 ± 4.2	0.79
102.	[11]	a pretreatment: 69.4 μg diflubenzuron	0.76 ± 0.24	14.2 ± 5.7	56.1 ± 2.3	0.85
103.	[11]	a US: 0 μg diflubenzuron	0.38 ± 0.11	4.9 ± 5.1	51.9 ± 3.5	0.89
104.	[11]	a US: 3.4 μg diflubenzuron	0.46 ± 0.13	−0.3 ± 3.2	28.8 ± 1.8	0.89
105.	[11]	a US: 8.5 μg diflubenzuron	4.35 ± 13.18	4.0 ± 3.8	27.2 ± 1.2	0.80
106.	[11]	a US: 16 μg diflubenzuron	0.14 ± 0.16	21.2 ± 4.0	46.9 ± 15.1	0.69
107.	[11]	a US: 32 μg diflubenzuron	0.62 ± 0.66	15.2 ± 6.7	30.3 ± 3.0	0.35
108.	[11]	a US: 69.4 μg diflubenzuron	2.3 ± 5.41	3.8 ± 11.2	25.4 ± 3.6	0.27

**Table 3:** Learning curve comparison. The significance level of each comparison is found below. Item numbers correspond to Table 1 and Table 2. Values in bold italics are considered significant, *p* < 0.05.

No	Agrochemicals compared	Significance level, *p*		
		B2	B3	B4
1.	item 1, a field dose of pymetrozine—item 2, 100× the field dose of pymetrozine	0.88	0.86	***2.3 × 10^−8^***
2.	item 1, a field dose of pymetrozine—item 3, sucrose only	***0.00035***	0.66	0.17
3.	item 2, 100× the field dose of pymetrozine—item 3, sucrose only	0.069	0.50	***0.00005***
4.	item 4, a field dose of pymetrozine—item 6, sucrose only	***0.054***	0.67	0.25
5.	item 7, 6.25% Bioganic—item 8, 1.56% Bioganic	0.89	0.98	***0.00008***
6.	item 7, 6.25% Bioganic—item 9, sucrose only	0.52	0.42	***6.5 × 10^−10^***
7.	item 8, 1.56% Bioganic—item 9, sucrose only	0.85	0.58	***0.00004***
8.	item 10, 6.25% Bioganic—item 12, sucrose only	***0.014***	0.87	***1.1 × 10^−14^***
9.	item 13, citronella odor—item 14, 6.25% Bioganic odor	0.088	***0.014***	***0.010***
10.	item 15, pignut—item 16, sweet fennel	0.14	0.89	***0.0001***
11.	item 17, 10 μL dicofol—item 18, sucrose only	0.15	***0.030***	***1 × 10^−8^***
12.	item 19, 10 μL dicofol—item 20, sucrose only	0.093	0.88	0.41
13.	item 21, butyric acid—item 22, DEET	***0.0076***	0.056	***0.00045***
14.	item 21, butyric acid—item 23, cinnamon	0.93	0.069	***0.014***
15.	item 22, DEET—item 23, cinnamon	***0.0085***	0.92	***3.2 × 10^−9^***
16.	item 24, citronella—item 25, cinnamon	0.18	0.94	0.18
17.	item 26, fluvalinate—item 27, acetone	0.094	***0.000014***	***0.0006***
18.	item 26, fluvalinate—item 28, flucythrinate	0.95	***0.007***	***0.0069***
19.	item 26, fluvalinate—item 29, cyfluthrin	0.99	***0.00005***	0.56
20.	item 26, fluvalinate—item 30, cypermethrin	0.89	0.23	0.37
21.	item 26, fluvalinate—item 31, permethrin	0.63	0.81	0.16
22.	item 26, fluvalinate—item 32, fenvalerate	0.96	***0.018***	0.66
23.	item 27, acetone—item 28, flucythrinate	0.36	***0.00001***	***0.0013***
24.	item 27, acetone—item 29, cyfluthrin	0.13	***1.3 × 10^−6^***	***0.015***
25.	item 27, acetone—item 30, cypermethrin	0.40	***0.001***	***0.012***
26.	item 27, acetone—item 31, permethrin	0.88	***0.008***	***0.0023***
27.	item 27, acetone—item 32, fenvalerate	0.20	***0.00003***	***0.010***
28.	item 28, flucythrinate—item 29, cyfluthrin	0.95	***0.0046***	0.061
29.	item 28, flucythrinate—item 30, cypermethrin	0.88	0.23	0.083
30.	item 28, flucythrinate—item 31, permethrin	0.64	***0.033***	0.055
31.	item 28, flucythrinate—item 32, fenvalerate	0.93	0.72	***0.033***
32.	item 29, cyfluthrin—item 30, cypermethrin	0.89	***0.0017***	0.82
33.	item 29, cyfluthrin—item 31, permethrin	0.63	***0.00044***	0.69
34.	item 29, cyfluthrin—item 32, fenvalerate	0.96	***0.0032***	0.86
35.	item 30, cypermethrin—item 31, permethrin	0.72	0.26	0.89
36.	item 30, cypermethrin—item 32, fenvalerate	0.92	0.38	0.67
37.	item 31, permethrin—item 32, fenvalerate	0.65	0.058	0.50
38.	item 33, water—item 34, acetone	0.76	0.99	0.87
39.	item 33, water—item 35, 0.01% coumaphos	0.76	0.99	0.85
40.	item 33, water—item 36, 1% coumaphos	0.65	0.93	0.49
41.	item 33, water—item 37, 10% coumaphos	0.77	0.99	0.83
42.	item 34, acetone—item 35, 0.01% coumaphos	0.93	0.97	0.93
43.	item 34, acetone—item 36, 1% coumaphos	0.97	0.97	0.72
44.	item 34, acetone—item 37, 10% coumaphos	0.92	0.99	0.75
45.	item 35, 0.01% coumaphos—item 36, 1% coumaphos	0.92	0.99	0.90
46.	item 35, 0.01% coumaphos—item 37, 10% coumaphos	0.99	0.99	0.76
47.	item 36, 1% coumaphos—item 37, 10% coumaphos	0.90	0.98	0.51
48.	item 38, water—item 39, acetone	0.70	0.92	0.60
49.	item 38, water—item 40, 0.005% diazinon	0.98	0.98	0.79
50.	item 38, water—item 41, 0.01% diazinon	0.98	0.99	0.78
51.	item 38, water—item 42, 0.025% diazinon	0.45	0.96	0.39
52.	item 39, acetone—item 40, 0.005% diazinon	0.98	0.98	0.79
53.	item 39, acetone—item 41, 0.01% diazinon	0.98	0.99	0.78
54.	item 39, acetone—item 42, 0.025% diazinon	0.45	0.96	0.39
55.	item 40, 0.005% diazinon—item 41, 0.01% diazinon	0.98	0.99	0.78
56.	item 40, 0.005% diazinon—item 42, 0.025% diazinon	0.45	0.96	0.38
57.	item 41, 0.01% diazinon—item 42, 0.025% diazinon	0.45	0.96	0.39
58.	item 43, hexane only—item 44, 0.05% diazinon	0.91	0.54	***0.046***
59.	item 43, hexane only—item 45, 07% coumaphos	***0.051***	0.98	0.63
60.	item 44, 0.05% diazinon—item 45, 0.7% coumaphos	***0.051***	0.97	0.62
61.	item 46, sucrose—item 47, endosulfan	0.83	0.99	***0.00017***
62.	item 46, sucrose—item 48, decis	0.81	0.96	0.08
63.	item 47, endosulfan—item 48, decis	0.81	0.96	0.077
64.	item 51, hexanal—item 52, endosulfan	0.78	0.66	0.75
65.	item 51, hexanal—item 53, decis	0.94	0.62	0.78
66.	item 51, hexanal—item 54, baytroid	0.92	0.47	0.84
67.	item 51, hexanal—item 55, sevin	0.86	0.72	0.71
68.	item 52, endosulfan—item 53, decis	0.70	0.27	0.96
69.	item 52, endosulfan—item 54, baytroid	0.92	0.47	0.84
70.	item 52, endosulfan—item 55, sevin	0.86	0.72	0.71
71.	item 53, decis—item 54, baytroid	0.99	0.16	0.90
72.	item 53, decis—item 55, sevin	0.92	0.48	0.53
73.	item 54, baytroid—item 55, sevin	0.90	0.96	0.53
74.	item 56, sucrose—item 57, endosulfan	0.54	0.80	***0.010***
75.	item 56, sucrose—item 58, decis	0.82	0.84	0.25
76.	item 56, sucrose—item 59, baytroid	0.92	0.09	***0.001***
77.	item 56, sucrose—item 60, sevin	0.59	0.41	***0.002***
78.	item 57, endosulfan—item 58, decis	0.55	0.90	***0.026***
79.	item 57, endosulfan—item 59, baytroid	0.64	0.52	***0.052***
80.	item 57, endosulfan—item 60, sevin	0.84	0.85	***0.006***
81.	item 58, decis—item 59, baytroid	0.94	0.13	***0.0038***
82.	item 58, decis—item 60, sevin	0.60	0.54	***0.0005***
83.	item 59, baytroid—item 60, sevin	0.64	0.29	0.51
84.	item 61, 0 μg tebufenozide—item 62, 16 μg tebufenozide	0.096	0.17	0.26
85.	item 61, 0 μg tebufenozide—item 63, 24 μg tebufenozide	***0.008***	0.90	***0.002***
86.	item 61, 0 μg tebufenozide—item 64, 32 μg tebufenozide	***6.5 × 10^−8^***	0.75	0.069
87.	item 61, 0 μg tebufenozide—item 65, 69.4 μg tebufenozide	***0.008***	0.07	0.56
88.	item 61, 0 μg tebufenozide—item 66, 131 μg tebufenozide	***0.036***	0.69	***2.8 × 10^−9^***
89.	item 62, 16 μg tebufenozide—item 63, 24 μg tebufenozide	***0.00015***	0.33	***0.0017***
90.	item 62, 16 μg tebufenozide—item 64, 32 μg tebufenozide	***0.00003***	0.52	0.086
91.	item 62, 16 μg tebufenozide—item 65, 69.4 μg tebufenozide	***3.5 × 10^−6^***	***0.0009***	0.24
92.	item 62, 16 μg tebufenozide—item 66, 131 μg tebufenozide	0.19	0.50	***2.3 × 10^−7^***
93.	item 63, 24 μg tebufenozide—item 64, 32 μg tebufenozide	0.52	0.85	0.94
94.	item 63, 24 μg tebufenozide—item 65, 69.4 μg tebufenozide	0.94	0.095	***0.0012***
95.	item 63, 24 μg tebufenozide—item 66, 131 μg tebufenozide	0.20	0.981	***0.036***
96.	item 64, 32 μg tebufenozide—item 65, 69.4 μg tebufenozide	0.48	0.09	***0.048***
97.	item 64, 32 μg tebufenozide—item 66, 131 μg tebufenozide	0.098	0.97	0.19
98.	item 65, 69.4 μg tebufenozide—item 66, 131 μg tebufenozide	0.16	***0.054***	***2.4 × 10^−7^***
99.	item 67, 0 μg tebufenozide—item 68, 16 μg tebufenozide	0.81	0.65	***1.4 × 10^−6^***
100.	item 67, 0 μg tebufenozide—item 69, 24 μg tebufenozide,	***0.032***	0.99	***1.8 × 10^−9^***
101.	item 67, 0 μg tebufenozide—item 70, 32 μg tebufenozide	***0.016***	0.75	***0.0062***
102.	item 67, 0 μg tebufenozide—item 71, 69.4 μg tebufenozide	***0.0046***	0.85	***1.3 × 10^−7^***
103.	item 67, 0 μg tebufenozide—item 72, 131 μg tebufenozide	***0.043***	0.47	***0.00005***
104.	item 68, 16 μg tebufenozide—item 69, 24 μg tebufenozide	0.17	0.64	0.15
105.	item 68, 16 μg tebufenozide—item 70, 32 μg tebufenozide	0.085	0.55	0.36
106.	item 68, 16 μg tebufenozide—item 71, 69.4 μg tebufenozide	0.08	0.77	0.29
107.	item 68, 16 μg tebufenozide—item 72, 131 μg tebufenozide	0.17	0.33	0.12
108.	item 69, 24 μg tebufenozide—item 70, 32 μg tebufenozide	0.38	0.75	0.078
109.	item 69, 24 μg tebufenozide—item 71, 69.4 μg tebufenozide	0.30	0.84	0.84
110.	item 69, 24 μg tebufenozide—item 72, 131 μg tebufenozide	0.90	0.45	***0.0025***
111.	item 70, 32 μg tebufenozide—item 71, 69.4 μg tebufenozide	0.92	0.67	0.12
112.	item 70, 32 μg tebufenozide—item 72, 131 μg tebufenozide	0.48	0.86	0.91
113.	item 71, 69.4 μg tebufenozide—item 72, 131 μg tebufenozide	0.44	0.40	***0.013***
114.	item 73, 0 μg diflubenzuron—item 74, 3.4 μg diflubenzuron	***0.028***	0.35	***4.5 × 10^−6^***
115.	item 73, 0 μg diflubenzuron—item 75, 8.5 μg diflubenzuron	***0.0006***	0.068	0.22
116.	item 73, 0 μg diflubenzuron—item 76, 16 μg diflubenzuron	***0.031***	0.075	0.30
117.	item 73, 0 μg diflubenzuron—item 77, 32 μg diflubenzuron	0.55	0.17	***4.8 × 10^−7^***
118.	item 73, 0 μg diflubenzuron—item 78, 69.4 μg diflubenzuron	***0.0004***	***0.0049***	***5.4 × 10^−6^***
119.	item 74, 3.4 μg diflubenzuron—item 75, 8.5 μg diflubenzuron	0.072	0.28	0.17
120.	item 74, 3.4 μg diflubenzuron—item 76, 16 μg diflubenzuron	0.84	0.31	***0.0028***
121.	item 74, 3.4 μg diflubenzuron—item 77, 32 μg diflubenzuron	0.48	0.55	0.11
122.	item 74, 3.4 μg diflubenzuron—item 78, 69.4 μg diflubenzuron	0.48	0.55	0.11
123.	item 75, 8.5 μg diflubenzuron—item 79, 16 μg diflubenzuron	0.17	0.96	0.55
124.	item 75, 8.5 μg diflubenzuron—item 77, 32 μg diflubenzuron	0.13	0.66	***0.046***
125.	item 75, 8.5 μg diflubenzuron—item 78, 69.4 μg diflubenzuron	0.86	0.85	***0.015***
126.	item 76, 16 μg diflubenzuron—item 77, 32 μg diflubenzuron	0.43	0.70	***0.0002***
127.	item 76, 16 μg diflubenzuron—item 78, 69.4 μg diflubenzuron	0.12	0.80	***0.00012***
128.	item 77, 32 μg diflubenzuron—item 78, 69.4 μg diflubenzuron	0.11	0.46	0.36
129.	item 79, 0 μg diflubenzuron—item 80, 3.4 μg diflubenzuron	0.87	0.27	***2.6 × 10^−10^***
130.	item 79, 0 μg diflubenzuron—item 81, 8.5 μg diflubenzuron	0.77	0.39	***2 × 10^−7^***
131.	item 79, 0 μg diflubenzuron—item 82, 16 μg diflubenzuron	***0.032***	0.26	0.40
132.	item 79, 0 μg diflubenzuron—item 83, 32 μg diflubenzuron	0.90	0.75	***0.0004***
133.	item 80, 3.4 μg diflubenzuron—item 81, 8.5 μg diflubenzuron	0.99	0.096	0.35
134.	item 80, 3.4 μg diflubenzuron—item 82, 16 μg diflubenzuron	0.66	0.83	0.42
135.	item 80, 3.4 μg diflubenzuron—item 83, 32 μg diflubenzuron	0.94	0.37	0.34
136.	item 81, 8.5 μg diflubenzuron—item 82, 16 μg diflubenzuron	0.42	0.095	0.53
137.	item 81, 8.5 μg diflubenzuron—item 83, 32 μg diflubenzuron	0.93	0.782	0.779
138.	item 82, 6 μg diflubenzuron—item 83, 32 μg diflubenzuron	0.59	0.35	0.58
139.	item 85, 0 μg tebufenozide—item 86, 16 μg tebufenozide	0.078	0.77	***0.043***
140.	item 85, 0 μg tebufenozide—item 87, 24 μg tebufenozide	***0.0053***	0.96	0.52
141.	item 85, 0 μg tebufenozide—item 88, 32 μg tebufenozide	***0.007***	0.71	***0.014***
142.	item 85, 0 μg tebufenozide—item 89, 69.4 μg tebufenozide	0.14	0.92	***0.0005***
143.	item 85, 0 μg tebufenozide—item 90, 131 μg tebufenozide	***0.033***	0.58	***0.00002***
144.	item 86, 16 μg tebufenozide—item 87, 24 μg tebufenozide	0.064	0.73	***0.039***
145.	item 86, 16 μg tebufenozide—item 88, 32 μg tebufenozide	0.12	0.92	0.13
146.	item 86, 16 μg tebufenozide—item 89, 69.4 μg tebufenozide	0.61	0.70	0.40
147.	item 86, 16 μg tebufenozide—item 90, 131 μg tebufenozide	0.58	0.82	***0.0033***
148.	item 87, 24 μg tebufenozide—item 88, 32 μgtebufenozide	0.90	0.68	***0.012***
149.	item 87, 24 μg tebufenozide—item 89, 69.4 μg tebufenozide	***0.012***	0.95	***0.0057***
150.	item 87, 24 μg tebufenozide—item 90, 131 μg tebufenozide	0.22	0.54	***0.00005***
151.	item 88, 32 μg tebufenozide—item 89, 69.4 μg tebufenozide	***0.031***	0.65	0.22
152.	item 88, 32 μg tebufenozide—item 90, 131 μg tebufenozide	0.33	0.91	0.61
153.	item 89, 69.4 μg tebufenozide—item 90, 131 μg tebufenozide	0.28	0.52	***0.0046***
154.	item 91, 0 μg tebufenozide—item 92, 16 μg tebufenozide	0.58	0.53	***0.0002***
155.	item 91, 0 μg tebufenozide—item 93, 24 μg tebufenozide	0.99	0.85	***0.00013***
156.	item 91, 0 μg tebufenozide—item 94, 32 μg tebufenozide	***0.0006***	0.25	***8.1 × 10^−6^***
157.	item 91, 0 μg tebufenozide—item 95, 69.4 μg tebufenozide	0.060	0.83	***1.8 × 10^−8^***
158.	item 91, 0 μg tebufenozide—item 96, 131 μg tebufenozide	0.16	***0.012***	***2.0 × 10^−7^***
159.	item 92, 16 μg tebufenozide—item 93, 24 μg tebufenozide	0.77	0.79	0.27
160.	item 92, 16 μg tebufenozide—item 94, 32 μg tebufenozide	0.17	0.98	0.40
161.	item 92, 16 μg tebufenozide—item 95, 69.4 μg tebufenozide	0.63	0.64	0.34
162.	item 92, 16 μg tebufenozide—item 96, 131 μg tebufenozide	0.65	0.78	0.084
163..	item 93, 24 μg tebufenozide—item 94, 32 μg tebufenozide	0.55	0.73	0.66
164.	item 93, 24 μg tebufenozide—item 95, 69.4 μg tebufenozide	0.56	0.94	0.65
165.	item 93, 24 μg tebufenozide—item 96, 131 μg tebufenozide	0.57	0.60	0.70
166.	item 94, 32 μg tebufenozide—item 95, 69.4 μg tebufenozide	***0.041***	0.42	0.98
167.	item 94, 32 μg tebufenozide—item 96, 131 μg tebufenozide	0.21	0.71	0.31
168.	item 95, 69.4 μg tebufenozide—item 96, 131 μg tebufenozide	0.98	0.35	0.25
169.	item 97, 0 μg diflubenzuron—item 98, 3.4 μg diflubenzuron	0.089	0.85	0.27
170.	item 97, 0 μg diflubenzuron—item 99, 8.5 μg diflubenzuron	0.80	0.76	0.058
171.	item 97, 0 μg diflubenzuron—item 100, 16 μg diflubenzuron	***0.034***	0.25	0.81
172.	item 97, 0 μg diflubenzuron—item 101, 32 μg diflubenzuron	0.27	0.72	0.32
173.	item 97, 0 μg diflubenzuron—item 102, 69.4 μg diflubenzuron	0.09	0.86	0.64
174.	item 98, 3.4 μg diflubenzuron—item 99, 8.5 μg diflubenzuron	0.099	0.64	0.15
175.	item 98, 3.4 μg diflubenzuron—item 100, 16 μg diflubenzuron	0.68	0.22	0.057
176.	item 98, 3.4 μg diflubenzuron—item 101, 32 μg diflubenzuron	0.38	0.62	0.97
177.	item 98, 3.4 μg diflubenzuron—item 102, 69.4 μg diflubenzuron	0.73	0.97	0.16
178.	item 99, 8.5 μg diflubenzuron—item 100, 16 μg diflubenzuron	***0.038***	0.37	***0.016***
179.	item 99, 8.5 μg diflubenzuron—item 101, 32 μg diflubenzuron	0.31	0.89	0.18
180.	item 99, 8.5 μg diflubenzuron—item 102, 69.4 μg diflubenzuron	0.096	0.63	***0.028***
181.	item 100, 16 μg diflubenzuron—item 101, 32 μg diflubenzuron	0.18	0.59	0.18
182.	item 100, 16 μg diflubenzuron—item 102, 69.4 μg diflubenzuron	0.42	0.19	0.59
183.	item 101, 32 μg diflubenzuron—item 102, 69.4 μg diflubenzuron	0.53	0.62	0.32
184.	item 103, 0 μg diflubenzuron—item 104, 3.4 μg diflubenzuron	0.64	0.40	***0.00002***
185.	item 103, 0 μg diflubenzuron—item 105, 8.5 μg diflubenzuron	0.77	0.89	***0.00009***
186.	item 103, 0 μg diflubenzuron—item 106, 16 μg diflubenzuron	0.23	***0.022***	0.75
187.	item 103, 0 μg diflubenzuron—item 107, 32 μg diflubenzuron	0.73	0.24	***0.0002***
188.	item 103, 0 μg diflubenzuron—item 108, 69.4 μg diflubenzuron	0.73	0.93	***0.00005***
189.	item 104, 3.4 μg diflubenzuron—item 105, 8.5 μg diflubenzuron	0.78	0.40	0.47
190.	item 104, 3.4 μg diflubenzuron—item 106, 16 μg diflubenzuron	0.14	***0.0005***	0.25
191.	item 104, 3.4 μg diflubenzuron—item 107, 32 μg diflubenzuron	0.82	0.063	0.67
192.	item 104, 3.4 μg diflubenzuron—item 108, 69.4 μg diflubenzuron	0.74	0.73	0.42
193.	item 105, 8.5 μg diflubenzuron—item 106, 16 μg diflubenzuron	0.76	***0.006***	0.23
194.	item 105, 8.5 μg diflubenzuron—item 107, 32 μg diflubenzuron	0.78	0.16	0.36
195.	item 105, 8.5 μg diflubenzuron—item 108, 69.4 μg diflubenzuron	0.89	0.99	0.64
196.	item 106, 16 μg diflubenzuron—item 107, 32 μg diflubenzuron	0.50	0.45	0.31
197.	item 106, 16 μg diflubenzuron—item 108, 69.4 μg diflubenzuron	0.70	0.18	0.20
198.	item 107, 32 μg diflubenzuron—item 108, 69.4 μg diflubenzuron	0.76	0.39	0.31

The second experiment investigated the effects of pymetrozine on complex Pavlovian conditioning where honey bees were trained to discriminate between two CSs, one of which was always paired with a US. A control group learned well (Table 1, item 6) and learning data fit well with the model (Table 2, item 6). A field dose of pymetrozine induced spread in the raw learning data (Table 1, item 4) and decreased the learning rate twice (Table 2, item 4) in comparison with the control group (Table 3, item 4). A pretreatment with 100× the field dose of pymetrozine changed the shape of the learning curve from exponential to quadratic function. In other words, the percent of conditioned responses increased during the first five trials then diminished to zero. Our model was not able to fit this learning curve (Table 2, item 5).

*Bioganic*. These experiments investigated the effects of Bioganic^®^ Lawn and Garden Spray Multi-Insect Killer (Bioganic Safety Brands, Roswell, GA, USA), on honey bee learning. This agrochemical is unique because it is composed almost entirely of thyme, clover, and sesame essential oils. A non-overlap procedure was used with a CS duration of 3 s, a US duration of 2 s and an intertrial interval of 10 min. Forager Africanized honey bees (*Apis mellifera* L.) were used. The bees were collected in glass vials from the sill of the laboratory colonies approximately 24 h prior to use. The vials were placed in an ice water bath to reduce activity and then placed in a harness, fed 1.8 M sucrose and set aside. The following day, each bee received a pre-test in which the antennae was stimulated with sucrose. If a vigorous proboscis response was observed, the bee was used 10 min or more later. The 10 min delay was used to reduce the excitation induced by sucrose stimulation. The agrochemical was administered orally and mixed with sucrose to make it palatable. Two doses were used. In one condition, a 1 μL droplet of 1.56% of Bioganic was used and in the other condition a 1 μL droplet of 6.25% of Bioganic was used [9].

In the first experiment, citronella was used as a CS and Bioganic was used as a US. The unconditioned stimulus in the control group was sucrose. In the control group, the number of conditioned responses increased to 65% (Table 1, item 9). The learning curve for the control group fit well with the model (Table 2, item 9). Bioganic 1.56% increased the number of conditioned responses during the initial four trials only; next, the number of responses decreased (Table 1, item 8). The model did not fit the learning data; R squared is 0.1859 (Table 2, item 8). Ability to learn (B4) was more than two times lower (Table 3, item 7) in comparison with the control group. Bioganic 6.25% was not able to serve as a US. Instead, it diminished the number of conditioned responses to zero (Table 1, item 7). The learning curve changed into a monotonically decreasing curve with ability to learn of 1.0 (Table 2, item 7). This coefficient B4 differed significantly in comparison with the control group (Table 3, item 6) and Bioganic 1.56% group (Table 3, item 5).

The second experiment with Bioganic used discrimination in bees with a CS+ of citronella, a CS− of cinnamon and a variable US. The number of conditioned responses in the control group increased to 75% at the end of learning session (Table 1, item 12). The model fit the learning data well (Table 2, item 12). Bioganic 1.56% destroyed learning in such a way that the percent of conditioned responses increased during the initial trials then diminished during the rest of training (Table 1, item 11). As a result, the learning data did not fit with the model (Table 2, item 11). Bioganic 6.25% very quickly decreased the conditioned responses to zero (Table 1, item 10). Thus, the learning curve became a monotonously decreasing curve (Table 2, item 10). Ability to learn differed significantly between the control group and Bioganic 6.25% group (Table 3, item 8). In the third experiment, citronella or 6.25% Bioganic odor was used as a CS+, with sucrose as the US. Bees were conditioned very well with each odor. During Trial 1 there were no conditioned responses with citronella (Table 1, item 13), but 30% CR with 6.25% Bioganic odor (Table 1, item 14). Both learning curves (citronella, (Table 2, item 13) and 6.25% Bioganic, (Table 2, item 14)) fit very well with the model. Ability to learn and readiness to learn were significantly higher with 6.25 Bioganic (Table 3, item 9).

*Comparison of pignut and sweet fennel as a CS*. Pignut (*Hyptis suaveolens* (L.) Poit) and sweet fennel (*Foeniculum vulgare* Mill) are essential oils that have been shown to control aphids [26]. As the use of essential oils to control insect pests increases, it is important to evaluate their effect on honey bee learning. A non-overlap procedure was used with a CS duration of 3 s, a US duration of 2 s and an intertrial interval of 10 min. Forager Africanized honey bees (*Apis mellifera* L.) were used. The bees were collected in glass vials from the sill of the laboratory colonies approximately 24 h prior to use. The vials were placed in an ice water bath to reduce activity and then placed in a harness, fed 1.8 M sucrose, and set aside. The following day, each bee received a pre-test in which the antennae was stimulated with sucrose. If a vigorous proboscis response was observed, the bee was used 10 min or more later. The 10 min delay was used to reduce the excitation induced by sucrose stimulation. The agrochemical was administered orally by stimulating the antennae with sucrose, and with the proboscis now extended, allowed to consume the dose. In the pignut condition, bees were fed a 1 μL droplet of 3.125% pignut oil. Bees in the sweet fennel condition were fed a 1 μL droplet of 3.125% fennel oil [15].

Bees readily associated both odors with a reward. The number of conditioned responses was higher with pignut (Table 1, item 15), though the number of CR with sweet fennel was also high (Table 1, item 16). Modeling revealed that ability to learn was higher with pignut (B4 = 92.1, Table 2, item 15) in comparison with sweet fennel (B4 = 80.9, Table 2, item 18) and this difference was significant (Table 3, item 10).

*Dicofol (Kelthane).* Dicofol is an acaricide that is chemically very close to DDT. It is generally considered harmless to honey bees, but has been shown to affect other insects by changing their natural behavior [27]. As in the previous investigations from the senior author’s laboratory, a non-overlap procedure was used with a CS duration of 3 s, US duration of 2 s and an intertrial interval of 10 min. Forager honey bees (*Apis mellifera* L.) were used. The bees were collected in glass vials from the sill of the laboratory colonies approximately 24 h prior to use. The vials were placed in an ice water bath to reduce activity and then placed in a harness, fed 1.8 M sucrose, and set aside. The following day, each bee received a pre-test in which the antennae was stimulated with sucrose. If a vigorous proboscis response was observed, the bee was used 10 min or more later. The 10 min delay was used to reduce the excitation induced by sucrose stimulation. The agrochemical was administered orally and mixed with sucrose to make it palatable. The dose consisted of a 10 μL droplet of 0.7 g/L of dicofol [16].

The first experiment studied the influence of dicofol on acquisition in bees with cinnamon as a CS. A control group pretreated with sucrose yielded up to 90% of conditioned responses (Table 1, item 18). Dicofol decreased the initial Trial 1 value as well as the maximum value of CR (Table 1, item 17). Accordingly, readiness to learn and ability to learn were higher in the control group (Table 2, item 18) than in the dicofol group (Table 2, item 17), with this difference being significant (Table 3, item 11). In the second experiment, bees were pretreated with dicofol or sucrose and taught to discriminate between the odors of cinnamon oil and a perfume. Only six trials were used in this experiment. Bees learned up to 75%–80% of CR with sucrose (Table 1, item 20) as well as with dicofol (Table 1, item 19). Though the coefficient B4 value was higher in the sucrose group (B4 = 90.2, Table 2, item 20) than in the dicofol group (B4 = 75.3, Table 2, item 19), this difference was not significant (Table 3, item 12). We hypothesize that six CS+ trials were not enough to assess the asymptotic value correctly. An insufficient number of trials led to an underestimated value of the learning rate and, subsequently, overestimation of the B4 value.

### 3.2. Agrochemicals Considered Repellent to Bees

*Butyric acid and DEET*. Butyric acid is considered to be an olfactory repellent to honey bees and is used to separate bees from honey by causing the bees to move away from honey combs [28]. DEET (*N,N*-diethyl-3-methylbenzamide) is one of the most widely used insect repellents. A non-overlap procedure was used with a CS duration of 3 s, a US duration of 2 s, and an intertrial interval of 10 min. Forager honey bees (*Apis mellifera caucasica*) were used. The bees were collected in glass vials at the laboratory feeder approximately 24 h prior to use. The vials were placed in an ice water bath to reduce activity and then placed in a harness, fed 1.8 M sucrose, and set aside. The following day, each bee received a pre-test in which the antennae was stimulated with sucrose. If a vigorous proboscis response was observed, the bee was used 10 min or more later. The 10 min delay was used to reduce the excitation induced by sucrose stimulation. The agrochemical was administered orally and mixed with sucrose to make it palatable. In one experiment, bees were fed a 1 μL droplet of 25% DEET and in the other a 1 μL droplet of 5.45 M of butyric acid [10].

In the first experiment, acquisition in bees was analyzed with a CS of butyric acid, DEET, or cinnamon. Bees readily associated each odor with a reward. With butyric acid, the number of CR was up to 90% (Table 1, item 21) and the learning curve fit very well with the model (Table 2, item 21). With DEET, the number of CR was up to 80% (Table 1, item 22) and the learning curve fit very well with the model (Table 2, item 22). With cinnamon, the number of CR was up to 95% (Table 1, item 23) and the learning curve fit very well with the model (Table 2, item 23). Ability to learn was the least with DEET in comparison with butyric acid (Table 3, item 13) and cinnamon (Table 3, item 15). At the same time, readiness to learn was higher with DEET in comparison with butyric acid.

*Citronella.* Citronella (*Cymbopogon winterianus* Jowitt) is an essential oil that has been suggested as a repellent for honey bees [29]. A non-overlap procedure was used with a CS duration of 3 s, a US duration of 2 s, and an intertrial interval of 10 min. Forager Africanized honey bees (*Apis mellifera* L.) were used. The bees were collected in glass vials from the sill of the laboratory colonies approximately 24 h prior to use. The vials were placed in an ice water bath to reduce activity and then placed in a harness, fed 1.8 M sucrose, and set aside. The following day, each bee received a pre-test in which the antennae was stimulated with sucrose. If a vigorous proboscis response was observed, the bee was used 10 min or more later. The 10 min delay was used to reduce the excitation induced by sucrose stimulation. Bees were exposed to the odor of a 1 μL droplet of 100% citronella oil [17].

Acquisition of the PER to a CS of cinnamon or a CS of citronella was compared. The maximum number of CR was 65% with citronella (Table 1, item 24) and 70% with cinnamon (Table 1, item 25). With citronella (Table 2, item 24) and cinnamon (Table 2, item 25), the learning curves fit well with the model, and no differences between coefficients were found (Table 3, item 16).

### 3.3. Sublethal Amounts of Agrochemicals Known to Be Harmful to Bees

*Fluvalinate, flucythrinate, cyfluthrin, cypermethrin, permethrin, fenvalerate.* These pesticides are pyrethroids that are lethal to honey bees in laboratory settings [30] but were tested at sublethal dosages to determine their effects on learning. The CS duration was 6 s, the US duration was 3 s, and an overlap procedure was used in which the US was presented 3 s after the CS was presented and both terminated together. The intertrial interval was 15 min. Eight training trials were given, but the first training trial was not reported in the original paper—*i.e*., data was presented only for trials 2–8 (7 trials). Unfortunately, no unpaired or discrimination control group was employed. Forager honey bees (*Apis mellifera* L.) were used. Bees exiting a colony were funneled into a closed 9 cm dia glass petri dish containing insecticide-treated filter paper where they remained for 24 h before testing. After exposure, the surviving bees were chilled, harnessed, and fed. They were tested 3 h later. Bees were exposed to LC_50_ dosage of fluvalinate (10.00 mg/dish), fenvalerate (1.00 mg/dish), permethrin (0.06 mg/dish), cypermethrin (0.10 mg/dish), cyfluthrin (0.10 mg/dish), flucythrinate (1.00 mg/dish) [18].

Acquisition was tested using a CS of thyme odor in bees pretreated with fluvalinate, flucythrinate, cyfluthrin, cypermethrin, permethrin, fenvalerate, or an acetone-only control. The data consisted of seven trials. The acetone-only control provided the most conditioned responses on Trial 1, the second actual trial (62.9%, Table 1, item 27). Other chemicals take up position in decreasing order as follows: permethrin (41.7%, Table 1, item 31), fluvalinate (38.5%, Table 1, item 26), cypermethrin (34.2%, Table 1, item 30), fenvalerate (26.7%, Table 1, item 32), flucythrinate (24.2%, Table 1, item 28), and cyfluthrin (2.9%, Table 1, item 29). Each learning curve fit well with the model. Ability to learn took the same position as the Trial 1 value.

Ability to learn positioned the chemicals as follows: acetone (91.8%, Table 2, item 27), fluvalinate (73.5%, Table 2, item 26), fenvalerate (70.4%, Table 2, item 32), cyfluthrin (68.6%, Table 2, item 29), cypermethrin (66.2%, Table 2, item 30), permethrin (65.0%, Table 2, item 31), and flucythrinate (46.7%, Table 2, item 28). In comparison with the control chemical acetone, readiness to learn and ability to learn were lower in fluvalinate (Table 3, item 17), flucythrinate (Table 3, item 23), cyfluthrin (Table 3, item 24), cypermethrin (Table 3, item 25), permethrin (Table 3, item 26), and fenvalerate (Table 3, item 27). In other words, these results show that these chemicals do really harm honey bees. Cyfluthrin was the most harmful for readiness to learn, and flucythrinate was the most harmful for ability to learn.

*Coumaphos.* Coumaphos is an organophosphate that has been used to control mite and beetle populations that infest beehives. The CS or US durations were not clear from the paper, or whether an overlap or non-overlapnon-overlap procedure was used. The intertrial interval was 10 min, and only 5 training trials were employed. No unpaired or discrimination control groups were employed. Forager honey bees of different ages (*Apis mellifera* L.) were used, as determined by wing shape. The chemical was applied to the dorsal thorax, or by intracranial injection, and left overnight. The bees were collected in glass vials from the sill of the laboratory colony located outside or from an indoor hive, chilled to reduce activity, and then harnessed and fed. Two hours after harnessing, bees received a pre-test and the bees that extended their proboscises were used for training [19].

Acquisition was studied in bees pretreated with an application to the thorax of water, acetone, 0.01% coumaphos, 0.1% coumaphos, or 10% coumaphos, with a CS of geraniol odor. Learning consisted of only five trials. In this experiment, zero conditioned responses were observed during the first trial with water (Table 1, item 33), acetone (Table 1, item 34), 0.01% coumaphos (Table 1, item 35), 0.1% coumaphos (Table 1, item 36), and 10% coumaphos (Table 1, item 37). The maximum number of CR for each chemical was in the range 55%–70%. Ability to learn was 70% with water (Table 2, item 33), 74.1% with acetone (Table 2, item 34), 77.5% with 0.01% coumaphos (Table 2, item 35), 82.0% with 0.1% coumaphos (Table 2, item 36), and 63.9% with 10% coumaphos (Table 2, item 37). The learning rate and ability to learn with 10% coumaphos were corrected. Due to the insufficient number of trials, the learning rate was underestimated and, subsequently, ability to learn was overestimated. No significant differences were found among this group of chemicals (Table 3, items 38–47).

*Diazinon.* Diazinon was tested in comparison with coumaphos as an organophosphate that is known to be harmful to bees [31]. The CS and US durations are not specified, and it is not clear whether an overlap or non-overlapnon-overlap procedure was used. The intertrial interval was 10 min, and only 5 training trials were employed. No unpaired or discrimination control groups were employed. Forager honey bees of different ages (*Apis mellifera* L.) were used, as determined by wing shape. The chemical was applied to the dorsal thorax, or by intracranial injection, and left overnight. The bees were collected in glass vials from the sill of the laboratory colony located outside or from an indoor hive, chilled to reduce activity, and then harnessed and fed. Two h after harnessing, bees received a pre-test and those bees that extended their proboscises were used for training [19].

Acquisition was studied in bees pretreated with an application to the thorax of water, acetone, 0.005% diazinon, 0.01% diazinon, or 0.025% diazinon, with a CS of geraniol odor. Learning consisted of only five trials. In this experiment, zero conditioned responses were observed during the first trial with water (Table 1, item 38), acetone (Table 1, item 39), 0.005% diazinon (Table 1, item 40), 0.01% diazinon (Table 1, item 41), and 0.025% diazinon (Table 1, item 42). The maximum number of CR was in the range 55%–78%. The learning rate and ability to learn were corrected with water (Table 2, item 38) and 0.01% diazinon (Table 2, item 41). No significant differences were found among this group of chemicals (see Table 3, items 48–57).

*Diazinon, coumaphos.* Acquisition was studied in bees pretreated with an intracranial injection of 1 μL of 0.005% diazinon in hexane, 0.07% coumaphos in hexane, or hexane alone, with a CS of geraniol odor. Learning consisted of only five trials. The maximum number of CR was 70% with hexane alone (Table 1, item 43), 30% with 0.005% diazinon (Table 1, item 44), and 65% with 0.07% coumaphos (Table 1, item 45). With hexane alone, due to an insufficient number of trials, the learning rate was underestimated and ability to learn was overestimated, so that these values were corrected (Table 2, item 43). With 0.07% coumaphos, the learning rate was the highest (Table 2, item 45) and differed significantly from hexane alone (Table 3, item 59) and 0.005% diazinon (Table 3, item 60). At the same time, ability to learn was lower with 0.005% diazinon in comparison with hexane alone (Table 3, item 58). Thus, diazinon was more harmful for bees than coumaphos.

*Endosulfan, decis, baytroid, sevin.* These insecticides were chosen because of their use to control the cotton boll weevil populations in Brazil [32]. The CS duration was 3 s, the US duration was 2 s, the intertrial interval was 10 min and a non-overlapnon-overlap procedure was used. Forager Africanized honey bees (*Apis mellifera* L.) were used. The bees were collected in glass vials from the sill of the laboratory colonies approximately 24 h prior to use. The vials were placed in an ice water bath to reduce activity and then placed in a harness, fed sucrose, and set aside. The following day, each bee received a pre-test in which the antennae was stimulated with sucrose. If a vigorous proboscis response was observed, the bee was used 10 min or more later. The 10 min delay was used to reduce the excitation induced by sucrose stimulation. The agrochemical was administered orally and mixed with sucrose to make it palatable [20].

In the first experiment, acquisition was studied in bees pretreated with 0.8 μL of endosulfan, 0.1 μL of decis, 0.3 μL of baytroid, 1.5 μL of sevin, or sucrose alone, with a CS of hexanal. Learning did not occur with baytroid (Table 1, item 49) and sevin (Table 1, item 50)—zero CR was observed during each trial. Practically no learning occurred with endosulfan—there were only four out of 12 trials with non-zero CR values (Table 1, item 47), thus the learning data did not fit with the model (Table 2, item 47). The maximum value of CR was 70% with sucrose (Table 1, item 46) and 50% with decis (Table 1, item 48). Decis impaired learning less than other chemicals from this group: there was no significant difference between its learning curve and the learning curve of sucrose (Table 3, item 62). In the second experiment, these chemicals were used as a CS and acquisition was studied in bees with a CS of hexanal plus 0.1 μL of endosulfan, decis, baytroid, sevin, or hexanal alone. Bees readily associated each odor with a reward. The maximum CR value was 75% with hexanal (Table 1, item 51), 65% with endosulfan (Table 1, item 52), 70% with decis (Table 1, item 53), 65% with baytroid (Table 1, item 54), and 75% with sevin (Table 1, item 55). Each learning curve fit well with the model (Table 2, items 51–55). No significant differences were found among these chemicals (Table 3, items 64–73).

In the third experiment, acquisition was studied in bees with a US of sucrose plus 0.1 μL endosulfan, decis, baytroid, sevin, or sucrose only. Learning was practically absent with baytroid (Table 1, item 59), so the learning curve became descendent (Table 2, item 59) and ability to learn was significantly lower in comparison with sucrose (Table 3, item 76). With sevin, learning was also absent (Table 2, item 60), so ability to learn was close to readiness to learn (Table 2, item 60) and significantly lower in comparison with sucrose (Table 3, item 77). With endosulfan, the number of CR initially increased, but from the ninth trial, its value progressively decreased (Table 1, item 57), so the fit with the model was not sufficient (Table 2, item 57) and ability to learn was significantly lower in comparison with sucrose (Table 3, item 74). Only decis supported learning, with a maximum number of CR of 55% (Table 1, item 58). This value was less than with sucrose (Table 1, item 56), but no significant differences between the model’s coefficients were found in comparison with sucrose (Table 3, item 75).

*Tebufenozide* (Confirm^®^2F). Tebufenozide is an insect growth regular. The CS duration was 3 s, the US duration was 2 s, the intertrial interval was 10 min and a non-overlapnon-overlap procedure was used. Honey bees (*Apis mellifera* L).were collected from the sill of the laboratory colony. No attempt was made to solely focus on forager bees. As a result, a mixture of forager, nest cleaning, and guard honey bees was used. The bees were collected in glass vials approximately 24 h prior to use. The vials were placed in an ice water bath to reduce activity and then the bees were placed in a harness, fed 1.8 M sucrose, and set aside. The following day, each bee received a pre-test in which the antennae was stimulated with sucrose. If a vigorous proboscis response was observed, the bee was used 10 min or more later. The 10 min delay was used to reduce the excitation induced by sucrose stimulation. The agrochemical was administered orally and mixed with sucrose to make it palatable [11].

In the first experiment, acquisition was studied in bees pretreated with a 10 μL solution containing 0 μg, 16 μg, 24 μg, 32 μg, 69.4 μg, or 131 μg of tebufenozide, and a CS of cinnamon. The maximum number of CR was 100% in the control group without tebufenozide (Table 1, item 61), 92% with 16 μg (Table 1, item 62), 76% with 24 μg (Table 1, item 63), 80% with 32 μg (Table 1, item 64), 100% with 69.4 μg (Table 1, item 65), and 64% with 131 μg (Table 1, item 66) of tebufenozide. Each learning curve fit well with the model (Table 2, item 61–66). With the exception of 16 μg (Table 3, item 84), each dose of tebufenozide significantly diminished the learning rate (Table 3, item 85–88). Ability to learn was decreased with a dose of 24 μg (Table 3, item 85) and 131 μg (Table 3, item 88). There were significant differences among the model’s coefficients with different doses of the chemical (Table 3, item 89, 90, 91, 92, 94, 95, 96, and 98).

In the second experiment, acquisition was studied in bees with a US of 1 μL of sucrose plus 0 μg, 16 μg, 24 μg, 32 μg, 69.4 μg, or 131 μg of tebufenozide, and a CS of cinnamon. The maximum number of CR was 92% in the control group without tebufenozide (Table 1, item 67), 80% with 16 μg (Table 1, item 68), 64% with 24 μg (Table 1, item 69), 88% with 32 μg (Table 1, item 70), 68% with 69.4 μg (table 1, item 71), and 76% with 131 69.4 μg of tebufenozide (Table 1, item 72). Each learning curve fit well with the model (Table 2, item 67–72). In each dose, tebufenozide significantly decreased ability to learn and, except for 16 μg, lowered the learning rate (Table 3, item 99–103). Ability to learn was significantly lower with 24 μg (Table 3, item 110) and 69.4 μg (Table 3, item 113) in comparison with 131 μg of tebufenozide.

In the third experiment, discrimination was studied in bees pretreated with a 10 μL solution containing 0 μg, 16 μg, 24 μg, 32 μg, 69.4 μg, or 131μg of tebufenozide, and a CS+ of cinnamon. The maximum number of CR was 88% in the control group without tebufenozide (Table 1, item 85), 77% with 16 µg (Table 1, item 86), 85% with 24 µg (Table 1, item 87), 65% with 32 µg (Table 1, item 88), 73% with 69.4 μg (Table 1, item 89), and 69% with 131 µg of tebufenozide (Table 1, item 90). Each learning curve fit well with the model (Table 2, item 85–90). The chemical significantly decreased ability to learn in the doses of 16 µg (Table 3, item 139), 32 µg (Table 3, item 141), 69.4 µg (Table 3, item 142), and 131 µg (Table 3, item 143) in comparison with the control group. The learning rate was significantly lower with 24 µg (Table 3, item 140), 32 µg (Table 3, item 141), and 131 µg (Table 3, item 143). Additionally, there were significant differences in ability to learn and the learning rate among different doses of the chemical (Table 3, item 144, 147, 148, 149, 150, 151, and 153).

In the fourth experiment, discrimination was studied in bees with a US of 1 μL of sucrose plus 0 μg, 16 μg, 24 μg, 32 μg, 69.4 μg, or 131 μg of tebufenozide, and a CS+ of cinnamon. The maximum number of CR was 88% in the control group without tebufenozide (Table 1, item 91), 77% with 16 µg (Table 1, item 92), 77% with 24 µg (Table 1, item 93), 60% with 32 µg (Table 1, item 94), 64% with 69.4 µg (Table 1, item 95), and 64% with 131 µg (Table 1, item 96). That said, with each dose, the number of CR reached its maximum value in the midst of the learning session then decreased. Each learning curve was fitted with the model (Table 2, item 91–96), though with the doses of 16 µg (Table 2, item 92), 24 μg (Table 2, item 93), 131 µg of tebufenozide (Table 1, item 96) a spread of CR values was found. The chemical significantly decreased ability to learn in each dose (Table 3, item 154–158) in comparison with the control group. The learning rate was significantly lower with 32 µg (Table 2, item 97). A significant difference in the learning rate was found between the doses of 32 µg and 69.4 µg (Table 3, item 166). No other differences in the model’s coefficients among doses were found.

*Diflubenzuron* (Dimilin^®^). Diflubenzuron is an insect growth regular that disrupts molting but has been shown to have no effect on honey bees [33]. The CS duration was 3 s, the US duration was 2 s, the intertrial interval was 10 min and a non-overlapnon-overlap procedure was used. Honey bees (*Apis mellifera* L.) were collected from the sill of the laboratory colony. No attempt was made to solely focus on forager bees. As a result, a mixture of forager, nest cleaning, and guard honey bees was used. The bees were collected in glass vials approximately 24 h prior to use. The vials were placed in an ice water bath to reduce activity and then the bees were placed in a harness, fed 1.8 M sucrose, and set aside. The following day, each bee received a pre-test in which the antennae was stimulated with sucrose. If a vigorous proboscis response was observed, the bee was used 10 min or more later. The 10 min delay was used to reduce the excitation induced by sucrose stimulation. The agrochemical was administered orally and mixed with sucrose to make it palatable [11].

In the first experiment, acquisition was studied in bees pretreated with a 10 μL solution containing 0 μg, 3.4 μg, 8.5 μg, 16 μg, 32 μg, or 69.4 μg of diflubenzuron, and a CS of cinnamon. The maximum number of CR was 92% in the control group without diflubenzuron (Table 1, item 76), 72% with 3.4 μg (Table 1, item 74), 80% with 8.5 μg (Table 1, item 75), 84% with 16 μg (Table 1, item 76), 72% with 32 μg (table 1, item 77), and 72% with 69.4 μg of diflubenzuron (Table 1, item 78). Each learning curve fit well with the model (Table 2, item 73–78). Each dose of the chemical significantly lowered the learning rate (Table 3, item 114, 115, 116, 118) with the exception of 32 μg (Table 3, item 117). Ability to learn was significantly decreased with 3.4 μg (Table 3, item 114), 32 μg (Table 3, item 117), and 69.4 μg (Table 3, item 118). There were also significant differences in ability to learn among different doses of the chemical (Table 3, item 120, 124, 125, 126, and 127).

In the second experiment, acquisition was in bees with a US of 1 μL of sucrose plus 0 μg, 3.4 μg, 8.5 μg, 16 μg, 32 μg, or 69.4 μg of diflubenzuron, and a CS of cinnamon. The maximum number of CR was 68% in the control group without diflubenzuron (Table 1, item 79), 36% with 3.4 μg (Table 1, item 80), 36% with 8.5 μg (Table 1, item 81), 44% with 16 μg (Table 1, item 82), 56% with 32 μg (Table 1, item 83), and 56% with 69.4 μg of the chemical (Table 1, item 84). The learning curve fit very well only in the control group (Table 2, item 79). Diflubenzuron increased the number of CR during some initial trials and decreased it during the second part of the session. Low R squared values were found for 3.4 μg (Table 2, item 80), 32 μg (Table 2, item 83), and especially 69.4 μg, for which the data did not fit with the model (Table 2, item 84). Ability to learn was significantly decreased with 3.5 μg (Table 3, item 129), 8.5 μg (Table 3, item 130), and 32 μg (Table 3, item 132). The learning rate was significantly lower with 16 μg in comparison with the control group (Table 3, item 131). There were no significant differences between the model’s coefficients among different doses (Table 3, item 133–138).

In the third experiment, discrimination was studied in bees pretreated with a 10 μL solution containing 0 μg, 3.4 μg, 8.5 μg, 16 μg, 32 μg, or 69.4 μg of diflubenzuron, and a CS+ of cinnamon. The maximum number of CR was 58% in the control group without diflubenzuron (Table 1, item 97), 69% with 3.4 μg (Table 1, item 98), 69% with 8.5 μg (Table 1, item 99), 62% with 16 μg (Table 1, item 100), 77% with 32 μg (Table 1, item 101), and 65% with 69.4 μg of the chemical (Table 1, item 102). Each learning curve fit well (Table 2, item 97–102). Ability to learn did not differ significantly between the control group and 3.4 μg of diflubenzuron (Table 3, item 169), 16 μg (Table 3, item 171), 32 μg (Table 3, item 172), and 69.4 μg (Table 3, item 173). The dose of 8.5 μg increased ability to learn on the trend level (Table 3, item 170, *p* = 0.058). With the dose of 16 μg, the learning rate was higher than in the control group (Table 3, item 171). A significant difference between ability to learn was found with some doses (Table 3, item 178, 180). The learning rate differed significantly between 8.5 μg and 16 μg of the chemical (Table 3, item 178).

In the fourth experiment, discrimination was studied in bees with a US of 1 μL of sucrose plus 0 μg, 3.4 μg, 8.5 μg, 16 μg, 32 μg, or 69.4 μg of diflubenzuron, and a CS+ of cinnamon. The maximum number of CR was 58% in the control group without diflubenzuron (Table 1, item 103), 35% with 3.4 μg (Table 1, item 104), 34% with 8.5 μg (Table 1, item 105), 42% with 16 μg (Table 1, item 106), 42% with 32 μg (Table 1, item 107), and 42% with 69.4 μg of the chemical (Table 1, item 108). With the dose of 69.4 μg of diflubenzuron, the number of CR increased during the initial trials, but decreased toward the end of the learning session (table 1, item 108). The learning curves fit well in the control group (Table 2, item 103), with 3.4 μg (table 2, item 104), with 8.5 μg (Table 2, item 105), and with 16 μg (Table 2, item 106). With the dose of 32 μg, the learning data were spread around the model curve (Table 2, item 107). With the dose of 69.4 μg, the learning curve looked like a sine wave that worsened fitting (Table 2, item 108). Ability to learn was decreased significantly with the doses of 3.4 μg (Table 3, item 184), 8.5 μg (Table 3, item 185), 32 μg (Table 3, item 187), and 69.4 μg (Table 3, item 188). The dose of 16 μg, instead, significantly decreased the learning rate (Table 3, item 186). Readiness to learn was higher with 16 μg in comparison with the control group (Table 3, item 186). Additionally, ability to learn was significantly higher with 16 μg in comparison with 3.4 μg (Table 3, item 190) and with 8.5 μg (Table 3, item 193).

## 4. Conclusions

This article deals with the effects on learning of several classes of agrochemicals, including: (1) those that are considered harmless to bees; (2) sublethal exposure to chemicals known to harm honey bees; and (3) putative repellents of honey bees.

*Pesticides known to harm honey bees*. Fluvalinate, flucythrinate, cyfluthrin, cypermethrin, permethrin, and fenvalerate worsened both readiness to learn and ability to learn. Experiments with a pretreatment of coumaphos or diazinon did not reveal harmful effects on learning. However, five trials did not allow us to examine learning in detail because some chemicals decreased the number of CR during the final trials when twelve trials were used. Such insecticides as endosulfan, decis, baytroid, and sevin were more harmful for bees. When used as a pretreatment, baytroid, sevin, and endosulfan impaired learning. The same effect was found when baytroid, sevin, or endosulfan was used as a US. Among those chemicals, decis was found to be less harmful in comparison with others. An organic pesticide, Bioganic, destroyed learning when used as a US. Thus, these harmful chemicals (pesticides) disturb the learning of bees as well.

*Repellents*. When citronella, sweet fennel, butyric acid, and DEET were used as a CS, no learning impairment was found, though these chemicals sometimes differed from each other. For example, the ability to learn was the least with DEET in comparison with butyric acid and readiness to learn was higher with DEET in comparison with butyric acid.

*Chemicals that are considered harmless to bees*. A pretreatment with a field dose of pymetrozine decreased the learning rate three times, and 100× the field dose practically prevented learning. A pretreatment with dicofol decreased readiness to learn and ability to learn. Some doses of tebufenozide, used as a pretreatment, a US, or in experiments with discrimination, decreased the learning rate and ability to learn in bees. A pretreatment with diflubenzuron decreased ability to learn and the learning rate in a dose dependent manner. When diflubenzuron was used as a US together with sucrose, ability to learn decreased in a dose dependent manner. However, pretreatment with the chemical did not affect learning in experiments with discrimination. Thus, those chemicals, even though they are considered harmless to bees, in fact, depending on dose, impair learning to a certain extent.

It should be noted that the first order system transfer function is not the only statistically approach to analyzing learning data. In considering PER learning in honey bees, Hartz, Ben-Shahar, and Tyler [34] proposed a model based on logistic growth curve analysis. A rationale for the use of the logistic growth curve model is that it is available in many statistical packages and no sophisticated programming is necessary. A comparison of the logistic growth curve model and the first order transfer model advocated here revealed that our model provided better fits for their data and that our model can extract more information from the data in the form of our three coefficients.

Our results suggest several recommendations.

*1. Employ models of the learning process in the design and analysis of learning experiments with agrochemicals*. Models provide information above and beyond simple significance testing. For example, in our model, the effect of the agrochemical on the insect’s “learning rate” (B2), “readiness to learn” (B3) and “ability to learn” (B4) can be independently assessed. Moreover, these coefficients can be compared across species and across various environmental contaminates.

We have used the model to characterize the learning of snails [35], rats [2], and humans [36]. We have also used the model to characterize learning deficits associated with multiple sclerosis [37], to evaluate patients with type 2 Diabetes Mellitus [22], and to examine the performance of children on the California Verbal Learning Test [23]. The use of mathematical models allows researchers to go beyond their individual data sets to guide research and to potentially reveal interesting relationships not readily seen with simple significance testing. For example, it would be of interest to study whether humans exposed to agrochemicals experience the same deficits in learning rate, ability to learn, or ability to learn as insects. 

Models can be used to provide recommendations for the selection of agrochemicals that minimize deleterious effects. Though the goal of insecticides is, in general, to kill insects, the question remains how these insecticides affect non-target organisms. For example, in our earlier application of the model [12] and in the present manuscript, the insect growth regulators tebufenozide (Confirm^®^2F) and diflubenzuron (Dimilin^®^) were examined for their effect on honey bee PER learning. It was recommended that Confirm^®^2F be used because, although it decreased the learning rate, it did not influence the ability to learn as did Dimilin^®^. As a second example, we applied the model to the influence of essential oils of sweet fennel and pignut on honey bees. These essential oils are used in Brazil to control aphids and the results from our model indicate that both are safe for honey bees.

*2. Increase the number of training trials*. To accurately model data, there must be a specific minimum number of training trials. For our model, the absolute minimum is six. In our laboratory, we use 12 training trials for simple Pavlovian conditioning where a conditioned stimulus is paired with an unconditioned stimulus. For complex conditioning in which the insect must form a discrimination between two conditioned stimuli—one of which is paired with an unconditioned stimulus (CS+) and the other is not (CS−), we use 12 training trials, 6 each with CS+ and CS−. When we modeled our own data in the course of this investigation, the analysis showed that 12 training trials may not be enough for experiments that examine discrimination because at six CS+ trials, we may be unable to determine ability to learn [16]. We now recommend that discrimination experiments involving agrochemicals use 24 trials consisting of 12 CS+ trials and 12 CS− trials. Such a recommendation would not have been possible without the application of a mathematical model.

*3. Employ unpaired and discrimination control groups*. Mathematical models of the learning process assume that the data under consideration is the result of a learned association. In the case of Pavlovian conditioning, the learned association is between a conditioned stimulus and unconditioned stimulus. Whether the association is learned can be easily detected with an unpaired control group (conditioned and unconditioned stimuli are not paired) or a discrimination group in which an organism responds differentially to the CS+ and CS−. It has always been surprising to the authors of this paper how so few agrochemical studies employ control groups to evaluate learning [8]. We believe that it is not enough for authors to refer to earlier studies that actually use control groups. Without control groups, it is impossible to assess that an agrochemical influences a learned association. It is just as plausible that the agrochemical increases sensitization, which would lead to pseudo-conditioning or, alternatively, produces habituation, which would lead to poor associative learning [38].

*4. The need for standardization of learning procedures and definitions for the testing of agrochemicals*. For a mathematical model of the learning process to be effective, it should be applied to procedures that are standardized across laboratories. Although progress has been made, there are no standardized procedures in the PER paradigm. Some researchers, for example, place bees in straw holders, some use metal tubes, and still others use plastic holders. Training variables related to the CS and US durations, intertrial intervals and stimulus intensity also vary from laboratory to laboratory [7]. The lack of standardization has not gone unnoticed in the honey bee research community and in response the COLOSS network has recently published a book of standard practices [7]. Though standardization of conditioning protocols is important, it is also important to precisely define learning phenomena. For what one laboratory calls Pavlovian conditioning, another may call alpha conditioning or even instrumental conditioning. Definitional issues related to learning phenomena still need to be addressed [39] and one way to highlight the importance of this issue is through the precision of mathematical models.

*5. Apply mathematical models to various conditioning procedures to provide an overall assessment of the agrochemical on learning.* Though the PER has become a standard method, new methods are being developed that can be used to assess the effects of agrochemicals on learning. These methods include operant conditioning [40] and various aversive conditioning protocols involved in escape, avoidance, and punishment [41,42]. By applying mathematical models to various conditioning protocols used to evaluate agrochemicals, the effect on target and non-target organisms can be better assessed.

In summary, we believe that the approach used to analyze this data offers a unique insight to the specific aspects of learning that are affected by exposure to agrochemicals. The application of a mathematical model allows us to sift through large pools of data to make more exact determinations. We hope the use of this model will inspire further research and help researchers fine tune their methods to draw the most efficient and accurate conclusions.
